# Supplementary material for: It's Getting Hot in Here: Piloting a Telemedicine OSCE Addressing Menopausal Concerns for Obstetrics and Gynecology Clerkship Students
Source: MedEdPORTAL. 2021 Apr 28;17:11146. doi: 10.15766/mep_2374-8265.11146 (PMC8079425; doi:10.15766/mep_2374-8265.11146)
Supplement: Supplementary file 1 — Preencounter Learner Instructions.docxStandardized Patient Case.docxPreencounter Learner Information (Door Card).docxPostencounter Learner Note Scoring Criteria.docxPostencounter Learner Note (Blank).docxPostencounter Learner Note (Example).docxPostencounter Standardized Patient Checklist.docx [file mep_2374-8265.11146-s001.zip › A. Preencounter Learner Instructions.docx]

Pre-Encounter Learner Instructions

*These instructions as well as step by step detail as to how to set up and run the required technology (Zoom) were provided to students via their learning management system, Sakai. We have omitted the technology instructions from this document as they differ institutionally, but recommend including this guidance for learners.*

**Orientation to the Ob/Gyn Standardized Patient Encounter**

The purpose of the Ob/Gyn CPX is to help to prepare you for Step 2 CS and residency. It is structured to be the SAME format as Step 2 CS. Cases are meant to represent cases you may have seen in clinic – for the purpose of this CPX, each case will be a patient who you are seeing in your outpatient clinic.

The experience is pass/fail. Don’t stress, but please be on time. We recommend that you log in a few minutes early to ensure that you are ready to access zoom through learning space at the time your encounter is scheduled to start.

For your patient encounter of day 1 of the CPX, when the case ends, you will have up to 15 minutes of verbal feedback with the standardized patient (SP). You will need to write your note in learning space once your verbal feedback ends.

The note you will write is the same format as Step 2 CS. Sample notes are annotated are here: <http://usmle.org/practice-materials/step-2-cs/patient-note.html>  Please review this note so you know what you will need to fill out after the session.

You will start with a door card of patient information on the computer for the patient encounter. This will not be available after you begin the encounter so please write the vitals down (if applicable). Time for the patient encounter will start immediately when you open the Zoom. Please keep track of your own time during the encounter as no time announcements will be given.

Each patient encounter is 15 minutes. In that time, you will need to take a focused history and counsel the patient on the plan of care. You will have 15 minutes to write a focused SOAP note for the patient after the interview. A count-down clock is also included on the post encounter portion letting you know how much time you have left. The setting of this case is a telehealth encounter.

Summary:

--This is meant to be helpful and practice for Step 2 CS

--The sequence of events is as follows: Door card, 15 min in the room, 15 min to write note

--If you finish before time is up, you can leave the room and work on the note. Once you leave you cannot go back in.

-- You will receive verbal feedback from the standardized patient after this encounter.
