# Supplementary material for: It's Getting Hot in Here: Piloting a Telemedicine OSCE Addressing Menopausal Concerns for Obstetrics and Gynecology Clerkship Students
Source: MedEdPORTAL. 2021 Apr 28;17:11146. doi: 10.15766/mep_2374-8265.11146 (PMC8079425; doi:10.15766/mep_2374-8265.11146)
Supplement: Supplementary file 1 — Preencounter Learner Instructions.docxStandardized Patient Case.docxPreencounter Learner Information (Door Card).docxPostencounter Learner Note Scoring Criteria.docxPostencounter Learner Note (Blank).docxPostencounter Learner Note (Example).docxPostencounter Standardized Patient Checklist.docx [file mep_2374-8265.11146-s001.zip › B. Standardized Patient Case.docx]

Standardized Patient Case

Date: March 2020

Primary Case Author: Sarah Dotters-Katz, MD, MMHPE

Secondary Case Author: Ben Harris, MD; Amy MacDonald, CNM

Standardized Patient Educator: Tracey Reynolds, BA

Name of Case: “It’s getting hot in here”

Name of educational and or assessment activity: Clinical Practice Examination

Patient Name: Lynette Springfield (she/her/hers)

Chief Complaint: Hot flashes

Most likely Diagnosis and Differential with rationale from history and/or physical exam:

| Most likely diagnosis: Menopause / perimenopause | Hot flashes |
| --- | --- |
|  | Vaginal dryness |
|  | Mood changes / irritability |
| Diagnosis #2: Hyperthyroidism | Hot flashes |
|  | Irregular periods |
| Diagnosis #3: Malignancy | Hot flashes |
|  | Age |
| Diagnosis #4: Anxiety Attack | Hot flashes |
|  | History of depression |

Challenge question/moment:

About 5 minutes into encounter – SP should have a hot flash, state “ugh, its happening again” and start fanning herself and appear uncomfortable.

Domains: Check all that apply

- Professionalism

X Communication and Interpersonal skills

X Medical History

- Physical exam
- Shared Decision Making

X Patient Education

X Clinical Reasoning

X Documentation

- Handoff
- Presentation
- Other:

Type and level of learner: Medical student in their clinical year

Case Objectives: please list specific objectives for each of the domains you have checked above:

By the end of this activity, learners will be able to:

1. Obtain a complete problem focused history as it pertains to gynecologic symptoms.
2. Perform an assessment of symptoms associated with hypoestrogenism.
3. Develop a complete differential diagnosis for menopausal symptoms.
4. The student will document a management plan and counsel patient regarding management of menopausal symptoms.
5. Demonstrate empathy to the concerns demonstrated by the standardized patient and express increased comfort conducting a medical interview and establishing therapeutic rapport virtually.

| SETTING: outpatient, in patient, ED, home, nursing home, rehab, group etc. | Outpatient telehealth encounter, she is a new patient |
| --- | --- |
| PATIENT PROFILE: Information about the “patient” that helps select an SP and helps the learner get an understanding of them as a person. SP will know more information about the patient than learner will ever ask but allows SP to portray a fully developed patient personality. If none of the items below are particulars for the case please write “all may be used.” | |
| Age range | Late 40’s to early 50’s |
| Religious/spiritual background | All may be used |
| Sex (e.g., male, female, intersex, transwoman, transman) | Female |
| Sexual Orientation (e.g., heterosexual, lesbian, gay, bisexual, pansexual, queer, asexual) | Heterosexual |
| Gender expression (e.g., man, woman, gender queer) | Woman (she/her/hers pronouns) |
| Race/ethnicity: | All may be used |
| Physical description (e.g., BMI, height range) | All may be used |
| Physical limitations | None are mentioned in the case, but an SP with any physical limitations may portray this character |
| Patient appearance (e.g., disheveled, hospital gown, business casual, casual) | Casual dress, SP should appear to be in a home environment from which she is connecting with the provider via a telehealth portal |
| Moulage + location (e.g., none, bruises, scars, body piercing, tattoos) | None |
| Affect (e.g., pleasant, cooperative) | Pleasant and chatty, however is frustrated with the symptoms she is experiencing and wants to be taken seriously |
| Family group (e.g., who is family, who they live with) | Lives with husband and 17yo son |
| Education | Bachelor’s degree |
| Level of health literacy | Middle—is familiar with anatomical words such as uterus or ovary and the role of estrogen as the “female hormone,” but is not aware of the hormonal axis e.g. |
| Employment, if any - present and past, noting any current stresses | First grade teacher |
| Home/homeless - type of dwelling, number of stories, owned or rented | Owns 2-bedroom 2 bath house |
| Financial situation- any current stresses | Middle class, no current stresses, is planning to help child pay for college |
| Insurance Status (e.g., un/under/insured, public/private, HMO/PPO) | Private insurance through her husband’s work |
| Habits (i.e., diet, exercise, caffeine, smoking, alcohol, drugs) | Tobacco: Denies  Alcohol, drugs, substances: Does not use drugs. Uses alcohol 1-2 times a week, glass of wine with dinner  **If asked – all alcohol abuse related questions negative  Diet: Vegetarian  Exercise: None  Caffeine: A cup of coffee in the morning, not in excess |
| Activities (i.e., hobbies, sports, clubs, friends) | All may be used |
| Typical day - what is the usual daily routine | Wakes up between 6-7am to get ready for school, breakfast generally on the go as she begins teaching at 7:30am. Returns home after school at 3pm and works on grading and lesson planning until her husband gets home at 6 or 6:30pm. They eat dinner together and usually watch a TV show before going to bed. |

| CASE INFORMATION | |
| --- | --- |
| Chief Concern: What the patient will say when greeted by the student. The patient’s primary reason for seeking medical care often stated in his/own words. | “I keep getting these horrible hot flashes” |
| Additional Concerns: Other, if any, concerns the patient has today (i.e., symptoms, requests, expectations, etc.) that will become part of set agenda. | None |
|  | |
| THE PATIENT STORY: The SP will be asked to tell their symptom story and the personal and emotion impact for each of their concerns. You will want to write this is the patient voice. The symptom story should be able to answer this question: “Tell me more about [chief concern/additional concern], starting at the beginning and bringing me up to now.”  The personal context should be able to answer questions concerning the broader personal/psychosocial context of symptoms, especially the patient beliefs/attributions.  The emotional context should be able to ask how are you doing with this, how does this make you feel, how has this affected you emotionally? IMPACT: How has this affected your life? How has this been for your family? | “Well I have been just getting these terrible hot spells for maybe the past 5 or so months. Sometimes they start when I go into a hotter room, or if I am teaching a lesson I am less familiar with so I am a bit nervous, but other times they can start seemingly out of nowhere. They are mostly uncomfortable, I hate feeling like I’m suddenly sweating, and they’re also embarrassing if I start like whipping off my jacket for no reason. It hasn’t really changed my life per se but it does bother me to get them so often and not be able to control them once it starts.” |
| HISTORY OF PRESENT ILLNESS: Although some of the HPI will be given in the patient’s symptom story, the learners will expand the story during the direct question section. Below describe the detailed history, usually about the chief concern, which the student must develop in order to make a useful assessment of the problem: | |
|  | |
| Onset (when; gradual or sudden) | They come on suddenly |
| Setting (what was going on or where was patient when symptoms first noticed?) | I got the first one right in the middle of teaching |
| Duration (how long) | Each one usually lasts about 3-4 minutes |
| Time relationships (frequency, constant or intermittent) | These started 4-5 months ago, they are intermittent |
| Location | My whole body gets hot |
| Radiation | They start in my chest and then spread out into my face, legs and arms until it is my whole body. |
| Quality | I feel sweaty all over, I often have to take off my sweater or my jacket. |
| Amount | It is really getting bothersome. I thought maybe it was a virus at first, but it has been going on for a few months now, and they seem to be happening more and more. I get one almost every day now. |
| Aggravated by what | Worse with alcohol (when I have a glass of wine), also get them during intercourse. They are more frequent at night - But I know it does happen almost every day. |
| Relieved by what | Taking clothes off, going outside if its cold, fanning myself. But – with time, it gets better on its own |
| Associated with what | Moving into warmer rooms, sex, alcohol, but also sometimes come on “out of the blue.” |
| Attitude (what does the patient think is the problem, and how does he/she feel about it) | These are really bothersome, I know it is probably nothing serious, but it is really affecting me at work. I imagine it has something to do with my “change of life,” but I am just hoping there is something we can do about it |
| Overall course | Started 4-5 months ago and becoming more frequent and bothersome |
| REVIEW OF SYSTEMS: Significant positives and negatives | |
| *Negative* | *Positive* |
| No fevers or chills | Difficulty sleeping, hot flashes awake from sleep. Difficulty falling asleep |
| No GI symptoms over the course of the month/no change in BM with menstrual cycle | Vaginal dryness with intercourse |
| No vaginal discharge | Mood changes—more irritable/short with her husband |
| No UTI symptoms (no pain or burning with urination, no urinary frequency or urgency |  |
| No heat or cold intolerance |  |
| No skin, hair or nail changes |  |
| No unintentional weight changes |  |
|  | |
| Past medical history |  |
| Medication allergies (Name and reaction) | None |
| Environmental allergies (Name and reaction) | None |
| Illnesses | Depression (not on meds, does see a therapist monthly, no SI/HI) |
| Vaccinations | Up to date, had flu shot this year |
| Surgeries | Delivery of 1 child by cesarean for breech presentation, D&C for miscarriage at ~6 weeks |
| Accidents/ injuries/ trauma | None |
| Hospitalization | Only for birth of child |
|  | |
| Inclusive sexual and reproductive history | |
| Sexual practices  Sexual partners  Protection: Use of safer sex practices  Use of birth control if appropriate  Risk of intimate partner violence | Vaginal and oral sex; does orgasm, currently content with sex life, although maybe has a bit less energy for sex than previously. Additionally, experiences some vaginal dryness with intercourse even when using OTC lubricant.  Currently 1 male partner, lifetime 3 male partners  None, “It’s just me and my husband”  None, “I’m too old for that!”  None, no domestic violence no sexual abuse |
| Ob/GYN HISTORY | Age of onset of menses: 15  Periods: Used to be regular, every 31 days, lasting 3-4 days, heavy on day 1. Now very irregular, has not had a period in 6-7 months.  Age of menopause: N/A, mother went through menopause at ~age 50  Pap smears: No history of abnormal pap smears. No cervical surgeries. Pap last year was normal and HPV negative  Hx of STDs: distant history of adequately treated trichomonas  Number of pregnancies: 2  Number of live births: 1  Number of miscarriages: 1  Number of abortions: 0 |
| Medications | Prescription/dose/reason: None  Over the counter/dose/reason: Motrin PRN/400mg/aches and pains  Herbs/supplements/dose/reason: Women’s Once A Day multivitamin  Other: None |
| Immunizations | X Tetanus  X Flu  X Hepatitis   - Pneumovax - HPV - Other |
| Tobacco products:   - Cigarettes - Cigar - Pipe - Chew - E-cigarettes | X Never   - Past- year started/year quit - Current   - Quantity   - # of years |
| Alcohol   - Beer - Wine - Liquor - Other | - Never - Past- year started/year quit   X Current   - - Quantity: 1-2 glasses of wine a week   - # of years: Past 15 years |
| Drugs   - Weed - Cocaine - Heroin - Meth - Other - IV - Inhalants - Other | X Never   - Past- year started/year quit - Current   - Quantity - # of years |
| Diet (describe) | Vegetarian, tries to eat healthy, but often has some sort of dessert after dinner |
| Exercise (describe) | Stands up in front of her class all day, will sometimes join in games at recess |
| List any other important social history or information important to this case | None |
| Family history |  |
| Mother, Father, Siblings, Grandparents, and other significant findings. | Family member, status (living age/deceased), medical conditions   - Father, deceased, type 2 diabetes, hypertension - Mother, deceased, type 2 diabetes - Sibling, 48 years old, Hypertension - Child, 17 years old, healthy |
|  |  |
| Physical Exam- List exam maneuvers expected for this case and any abnormal findings that SP will simulate. (tenderness, hyper-hypo reflex, rebound, weakness etc. )  N/A | |
| PHYSICAL EXAM FINDINGS |  |
| 1. Written in layman’s terms | N/A |
| 1. General appearance- affect, appearance, position of patient at opening (i.e. sitting, laying down, holding abdomen etc.) | Well appearing female sitting up and in no acute distress |
| 1. Vital signs | N/A |
| 1. Specific findings and affect | N/A |
| 1. Response to certain physical movements | N/A |
|  |  |
| DIAGNOSIS AND DIFFERENTIAL |  |
| Diagnosis with support from positive and negative history and PE findings | Menopause/perimenopause: hot flashes, vaginal dryness, mood changes/irritability, patient age |
| Differential with support from positive and negative history and PE findings | **Hyperthyroidism**: hot flashes, irregular periods, mood changes, no other constitutional changes (skin, hair, nails, weight e.g.) make this less likely  **Malignancy**: hot flashes, age, not associated with other systemic symptoms make this less likely, but would not want to miss this  **Anxiety attack**: hot flashes, history of depression, no recent history of social or life stress make this less likely  **Pregnancy:** hot flashes, secondary amenorrhea, age and duration make this less likely  **Pheochromocytoma**: hot flashes, unlikely as no other constitutional symptoms, rare disease  **ETOH abuse**: hot flashes, unlikely due to low consumption, CAGE negative  **Dietary choices (spicy food e.g.):** frequency/duration make this unlikely, not associated with eating  **Infection**: hot flashes (could be fever), frequency and duration make this less likely, not associated with other infectious or UTI symptoms |
|  |  |
| MANAGEMENT OR DIAGNOSTIC PLAN | Labs: TSH, FSH, pregnancy test  Counseling: lifestyle changes – weight loss, dressing in layers, lowering room temp, avoiding triggers, validation of severity of symptoms  Treatment: offer SSRI for tx of hot flashes, offer HRT (must be estrogen and prog) – must have risk / benefit discussion, vaginal estrogen for dryness |
| PROFESSIONALISM ISSUES OR CHALLENGES: | Student should demonstrate empathy to patient complaint and provide supportive statements when SP experiences a hot flash during the appointment. |
