# Supplementary material for: It's Getting Hot in Here: Piloting a Telemedicine OSCE Addressing Menopausal Concerns for Obstetrics and Gynecology Clerkship Students
Source: MedEdPORTAL. 2021 Apr 28;17:11146. doi: 10.15766/mep_2374-8265.11146 (PMC8079425; doi:10.15766/mep_2374-8265.11146)
Supplement: Supplementary file 1 — Preencounter Learner Instructions.docxStandardized Patient Case.docxPreencounter Learner Information (Door Card).docxPostencounter Learner Note Scoring Criteria.docxPostencounter Learner Note (Blank).docxPostencounter Learner Note (Example).docxPostencounter Standardized Patient Checklist.docx [file mep_2374-8265.11146-s001.zip › C. Preencounter Learner Information (Door Card).docx]

**Pre-Encounter Learner Information (Door Card)**

**Instructions to the Student**:

**Patient Information**

| **Patient Name:**Lynette Springfield  **Setting:**  Outpatient clinic, Telehealth Visit  **Patient Information:**  Mrs. Springfield (she/her) is a 51-year-old female who has presented as a new patient to your clinic for hot flashes  **Vitals: This is a telehealth encounter – no vitals obtained** |
| --- |

**Your Task**

| In the ***15 minutes*** with the patient:              Obtain problem-focused history that includes and Ob/Gyn history.   In the remaining ***15 minutes***:             Write a note that includes your medical decision-making, including a prioritized differential and a proposed workup/management plan. |
| --- |
