# Supplementary material for: It's Getting Hot in Here: Piloting a Telemedicine OSCE Addressing Menopausal Concerns for Obstetrics and Gynecology Clerkship Students
Source: MedEdPORTAL. 2021 Apr 28;17:11146. doi: 10.15766/mep_2374-8265.11146 (PMC8079425; doi:10.15766/mep_2374-8265.11146)
Supplement: Supplementary file 1 — Preencounter Learner Instructions.docxStandardized Patient Case.docxPreencounter Learner Information (Door Card).docxPostencounter Learner Note Scoring Criteria.docxPostencounter Learner Note (Blank).docxPostencounter Learner Note (Example).docxPostencounter Standardized Patient Checklist.docx [file mep_2374-8265.11146-s001.zip › D. Postencounter Learner Note Scoring Criteria.docx]

**Post Encounter Learner Note Scoring Criteria: Lynette Springfield (hot flashes)**

**Patient History**

*Place a 1 in the points column for each aspect of the patient history included in the note. Then sum all the points to get the point total for the patient history.*

| **Aspect of History** | **1 pt per item** |
| --- | --- |
| Location: starts in chest, spreads all over |  |
| Quality: hot and sweaty all over |  |
| Severity: moderate |  |
| Onset: 4-5 months ago |  |
| Duration: 3-4 minutes, sudden onset, slow offset |  |
| Mother had menopause about this time (age 50) |  |
| Associated symptoms: Vaginal dryness |  |
| Associated symptoms: irritability / mood changes |  |
| Associated symptoms: Sleep changes |  |
| Exacerbating features: Alcohol, sex, moving into warmer spaces |  |
| Timing: More at night |  |
| Non-smoker |  |
| Frequency – almost every day |  |
| *Total number of points* |  |

**Physical Exam**

*Place a 2 in the points column for each component of the virtual physical exam included in the note. Then sum all the points to get the point total for the physical exam.*

| **Component of Physical Exam** | **2 pts per item** |
| --- | --- |
| Explicitly note – NO vitals as this is telehealth |  |
| General appearance |  |
| *Total number of points* |  |

**Differential Diagnosis**

*For leading diagnoses give 1 point for diagnosis and 1 point for supporting data place either a “1” or a “2” in the points column depending on what is included in the note. For less likely diagnoses only give the full point if the diagnosis and reasoning for why it is less likely is included, otherwise give no points. Do not give points for a leading diagnosis included in the less likely diagnoses section. Sum the number of all the points to get the point total for the differential diagnosis section.*

| **Leading Diagnoses** | | |
| --- | --- | --- |
| **Diagnosis** | **Supporting Data** | **1 pt for dx 1pt for supporting data (max 2 pts per dx)** |
| Menopause/perimenopause | +Hot flashes  +Mood changes  +Vaginal dryness  +patient age |  |
| Hyperthyroidism | +Hot flashes  +mood changes  +irregular periods  -no h/o thyroid disease |  |
| Malignancy | +hot flashes  +patient age |  |
| Anxiety | +hot flashes  -no anxiety history  -no social stress/life stress |  |
| **Other Diagnoses to Consider** | | |
| **Diagnosis** | **Reason less likely** | **1 pt for dx and reasoning**  **(max 1 pt per dx)** |
| Pregnancy | -age  -no other associated symptoms |  |
| Infection | -time course |  |
| Pheochromocytoma | -rare diagnosis  -no other associated symptoms (palpitations, blood pressure changes, weight loss) |  |
| ETOH abuse | -CAGE negative, rare ETOH consumption |  |
| Dietary choices (MSG use, Spicy foods) | -no link to consumption of foods |  |
| *Total number of points* | |  |

**Diagnostic Work-Up and Management/Counseling**

*For high yield diagnostics and management/counseling strategies give 2 points per item included in the note. For appropriate diagnostics and management/counseling strategies give 1 point per item included in the note. Sum all points to get point total for diagnostic work-up and Management/Counseling.*

| **High Yield** | |
| --- | --- |
| **Test or Management Strategy** | **2 points per item** |
| TSH or TFTs |  |
| FSH level |  |
| Pregnancy test |  |
| Discussion of lifestyle changes (must list at least one – weight loss, lowering room temperature, dressing layers, avoiding triggers) |  |
| Validation of severity of symptoms |  |
| Discuss / Offer SSRI or SNRI |  |
| Discuss / Offer HRT (must say estrogen and progesterone) |  |
| **Appropriate** | |
| **Test or Management Strategy** | **1 point per item** |
| LH level |  |
| Estrogen level |  |
| Cancer labs (CA-125 e.g.) |  |
| Discuss/ Offer topical estrogen for vaginal dryness |  |
| *Total number of points* |  |

**Total score:**

| **Component** | **Potential Points** | **Formative Score Guidance** |
| --- | --- | --- |
| History | X/13 | **Excellent=**10-13 points  **Good=** 8-9 points  **Fair=** 7-6 points  **Concerning=** 4-5 points  **Very concerning=** 0-3 points |
| Physical Exam | X/4 | **Excellent:** 4 points  **Fair**: 2 points  **Concerning:** 0 points |
| Differential Diagnosis | X/10 | **Excellent=** 10 points  **Good=** 8-9 points  **Fair=** 7-6 points  **Concerning=** 4-5 points  **Very concerning=** 0-3 points |
| Diagnostic Work Up/Management | X/18 | **Excellent** >15 points  **Good:** 10-14 points  **Fair** 6-9 points  **Concerning:** <6 points |
| Total | X/45 | **Excellent:** ≥39 points  **Good:** 38-29 points  **Fair:** 28-20 points  **Concerning:** 20-13 points  **Very Concerning:** <13 points |
