# Supplementary material for: It's Getting Hot in Here: Piloting a Telemedicine OSCE Addressing Menopausal Concerns for Obstetrics and Gynecology Clerkship Students
Source: MedEdPORTAL. 2021 Apr 28;17:11146. doi: 10.15766/mep_2374-8265.11146 (PMC8079425; doi:10.15766/mep_2374-8265.11146)
Supplement: Supplementary file 1 — Preencounter Learner Instructions.docxStandardized Patient Case.docxPreencounter Learner Information (Door Card).docxPostencounter Learner Note Scoring Criteria.docxPostencounter Learner Note (Blank).docxPostencounter Learner Note (Example).docxPostencounter Standardized Patient Checklist.docx [file mep_2374-8265.11146-s001.zip › E. Postencounter Learner Note (Blank).docx]

**Name:_________________________________________ Date:___________________**

**Post-Encounter Learner Note**

| HISTORY: Describe the history you just obtained from this patient. Include only information (pertinent positives and negatives) relevant to this patient's problem(s). | |
| --- | --- |
| PHYSICAL EXAMINATION: Describe any positive and negative findings relevant to this patient's problem(s). Be careful to include only those parts of examination you performed in this encounter. | |
| DATA INTERPRETATION: Based on what you have learned from the history and the physical examination, list up to 3 diagnoses that might explain this patient's complaint(s). List your diagnoses from most to least likely. For some cases, fewer than 4 diagnoses will be appropriate. Then, enter the positive or negative findings from the history and the physical examination (if present) that support each diagnosis. Lastly, list initial diagnostic studies (if any) you would order for each listed diagnosis (e.g. restricted physical exam maneuvers, laboratory tests, imaging, ECG, etc.) and anything you would offer as management. | |
| Diagnosis #1: | |
| History Finding(s) | |
|  | |
|  | |
|  | |
| Diagnosis #2: | |
| History Finding(s) | |
|  | |
|  | |
|  | |
| Diagnosis #3: | |
| History Finding(s) | |
|  | |
|  | |
|  | |
| Diagnosis #4: | |
| History Finding(s) | |
|  | |
|  | |
|  | |
| Other Diagnoses to Consider (please note why these are less likely): | |
| Other Diagnoses | Why less likely? |
|  |  |
|  |  |
|  |  |
|  |  |
| Work up/Diagnostics | Why do this? |
|  |  |
|  |  |
|  |  |
| Management / Counseling | Why do this? |
|  |  |
|  |  |
|  |  |
|  |  |
