# Supplementary material for: It's Getting Hot in Here: Piloting a Telemedicine OSCE Addressing Menopausal Concerns for Obstetrics and Gynecology Clerkship Students
Source: MedEdPORTAL. 2021 Apr 28;17:11146. doi: 10.15766/mep_2374-8265.11146 (PMC8079425; doi:10.15766/mep_2374-8265.11146)
Supplement: Supplementary file 1 — Preencounter Learner Instructions.docxStandardized Patient Case.docxPreencounter Learner Information (Door Card).docxPostencounter Learner Note Scoring Criteria.docxPostencounter Learner Note (Blank).docxPostencounter Learner Note (Example).docxPostencounter Standardized Patient Checklist.docx [file mep_2374-8265.11146-s001.zip › F. Postencounter Learner Note (Example).docx]

**Name:____Medical Student______________________ Date:________XX/XX/XXXX________**

**Post-Encounter Learner Note**

| HISTORY:  CC: “These hot flashes are driving me crazy”  HPI: 51yo G2P1011 with LMP >6 months ago, presents with hot flashes. They have been increasing over the last few months, but really only started 4-5 months ago. They come on suddenly out of the blue. They start in the patient’s chest, then move up into the face and down through lower extremities. They last about 3-4 minutes and ease away. Alcohol, sex seem to bring them on, and the patient has more at night. Time makes them better. She has noted increased vaginal dryness, more irritability, and maybe less energy for sex. Mother went through menopause at age 50.  She denies fevers, chills, heat or cold intolerance, changes to skin or nails, and weight loss. She denies any urinary frequency or urgency, and any GI symptoms. She does not note any change in her vaginal discharge.  PMh: depression  PSh: c/s, D+C  OB: G2P1011 🡪 CS x1 for breech, SAB x1  Gyn: menarche at 15, periods were regular – every 31 days lasting 3-4 days, with one heavy day. Has not bled in 6-7 months. Distant history of STD (trichomonas), no abnormal paps smears, most recent was normal and HPV negative. Sexually active with husband only (3 lifetime partners, other 2 were in teens). Usually has orgasms, notes mild vaginal dryness over last few months as above.  Soc: Works as a 1^st^ grade teacher, married and lives with partner and 17yo son. No tobacco, denies recreational drugs, does have 1 glass of wine 2-3 days/week  Immunizations: flu up to date  Fhx: mom and dad with DM, sibling with HTN | |
| --- | --- |
| PHYSICAL EXAMINATION: Describe any positive and negative findings relevant to this patient's problem(s). Be careful to include only those parts of examination you performed in this encounter.  Vitals: Not given, telehealth encounter  Gen: Well appearing overweight female in no acute distress | |
| DATA INTERPRETATION: Based on what you have learned from the history and the physical examination, list up to 3 diagnoses that might explain this patient's complaint(s). List your diagnoses from most to least likely. For some cases, fewer than 3 diagnoses will be appropriate. Then, enter the positive or negative findings from the history and the physical examination (if present) that support each diagnosis. Lastly, list initial diagnostic studies (if any) you would order for each listed diagnosis (e.g. restricted physical exam maneuvers, laboratory tests, imaging, ECG, etc.) | |
| Diagnosis #1: Menopause / perimenopause | |
| History Finding(s): | |
| Hot flashes | |
| Vaginal dryness | |
| Mood changes / irritability | |
| Diagnosis #2: Hyperthyroidism | |
| History Finding(s): | |
| Hot flashes | |
| Irregular periods | |
| No skin, nail or hair changes (-) | |
| Diagnosis #3: Malignancy | |
| History Finding(s): | |
| Hot flashes | |
| Age | |
| Diagnosis #4: Anxiety attack | |
| History Finding(s): | |
| Hot flashes | |
| Depression history | |
| Other Diagnoses to Consider (please note why these are less likely): | |
| Pregnancy | Age, time frame |
| Infection | Time frame |
| Pheochromocytoma | Normal BP, no weight loss, rare disease |
| ETOH abuse | Rare etoh use, CAGE negative |
| Dietary choices (MSG use, Spicy foods) | Time frame, other sx (vaginal dryness) |
| Management / Work up / Counseling | Why do this? |
| Labs: TSH, FSH, pregnancy test | Can r/o thyroid disease and pregnancy, FSH can help dx menopause although this can also be a clinical diagnosis |
| Counseling: life style changes – weight loss, dressing in layers, lowering room temp, avoiding triggers, validation of severity of symptoms | Non-invasive, low cost, easy to do with potential to change severity of symptoms |
| Treatment: offer SSRI or SNRI for tx of hot flashes | Best non-hormonal option |
| Treatment: offer HRT, estrogen and progesterone (uterus is intact), with risk benefit discussion | Good treatment for hot flashes, but has risks and benefits |
