# Supplementary material for: It's Getting Hot in Here: Piloting a Telemedicine OSCE Addressing Menopausal Concerns for Obstetrics and Gynecology Clerkship Students
Source: MedEdPORTAL. 2021 Apr 28;17:11146. doi: 10.15766/mep_2374-8265.11146 (PMC8079425; doi:10.15766/mep_2374-8265.11146)
Supplement: Supplementary file 1 — Preencounter Learner Instructions.docxStandardized Patient Case.docxPreencounter Learner Information (Door Card).docxPostencounter Learner Note Scoring Criteria.docxPostencounter Learner Note (Blank).docxPostencounter Learner Note (Example).docxPostencounter Standardized Patient Checklist.docx [file mep_2374-8265.11146-s001.zip › G. Postencounter Standardized Patient Checklist.docx]

Standardized Patient CPX Student Scoring Criteria: Menopause/Perimenopause

**Post-Encounter Standardized Patient Checklist for Students**

|  | Poor | Fair | Adequate | Very Good | Excellent |
| --- | --- | --- | --- | --- | --- |
| 1. The student in general created an environment where I felt safe talking about the menopausal symptoms I was experiencing. | ( ) | ( ) | ( ) | ( ) | ( ) |
| 2.The student’s non-verbal (body language, mannerisms etc) created a comfortable environment for me to talk openly about my concerns regarding my symptoms. | ( ) | ( ) | ( ) | ( ) | ( ) |
| 3.The student asked questions in a way that created a comfortable environment for me to talk openly about my concerns regarding my symptoms. | ( ) | ( ) | ( ) | ( ) | ( ) |

**Interpersonal**

| Interpersonal skills reflect a student’s ability to provide an effective exchange of information and develop a therapeutic relationship with their patients. They are a combination of communication skills (questioning and information-sharing) and relational skills (empathy and respect). These skills are particularly important to assess in the context of a telemedicine encounter where the use of technology may make it more difficult for students to convey appropriate warmth and empathy. The components of interpersonal skills being evaluated in this exam are: | | | | | |
| --- | --- | --- | --- | --- | --- |
| 4.  Introduction | ( )  Poor: *Does not introduce self *Does not identify you by name *Disinterested greeting | ( )  Fair | ( )  Adequate: *Introduces self *Identifies you by name *Appropriate greeting | ( )  Very Good | ( )  Excellent: *Introduces self by name and position *Identifies you by name *Warm and engaging greeting |
| 5.  Questioning Skills | ( )  Poor: *No use of open-ended questions *Multiple interruptions *Scattered and disjointed line of questioning | ( )  Fair | ( )  Adequate: *Some use of open-ended questions *Few interruptions *Basic flow to line of questioning | ( )  Very Good | ( )  Excellent: *Effective use of multiple open-ended questions *Zero to minimum interruptions *Smooth transitions and natural flow |
| 6.  Elicit Patient Perspective | ( )  Poor: *Uninterested in your explanatory model *Unconcerned with how illness may affect well-being *Resistant to incorporating your viewpoints into decision making | ( )  Fair | ( )  Adequate: *Acknowledges your explanatory model *Receptive to your concerns over impact of illness on well-being *Incorporates your input into decision making | ( )  Very Good | ( )  Excellent: *Proactively assesses your explanatory model *Explores your concerns over impact of illness on well-being *Proactively solicits your viewpoint in decision making |
| 7.  Verbal Communication | ( )  Poor: *Uses medical jargon excessively *Thoughts consistently disorganized *Tone of speech conveys indifference or detachment | ( )  Fair | ( )  Adequate: *Limited use of medical jargon *Most thoughts are well organized *Comfortable tone of speech | ( )  Very Good | ( )  Excellent: *Avoids medical jargon or readily explains it *Thoughts are consistently well organized and easy to understand *Uses warm and accepting tone of speech |
| 8.  Non-verbal Communication | ( )  Poor: *Unable to make eye contact *Awkward physical distance, facial expressions, or touching *Conveys disinterest or apathy *Appears uncomfortable with the digital format | ( )  Fair | ( )  Adequate: *Maintains some eye contact *Appropriate physical distance, facial expressions *Conveys interest and concern *Navigates the digital format appropriately | ( )  Very Good | ( )  Excellent: *Consistent eye contact *Uses physical distance, facial expressions, or touching effectively *Conveys attentiveness and compassion *Navigates digital environment gracefully, ensures you are comfortable using technology for the medical encounter |
| 9.  Empathy | ( )  Poor: *Ignores or fails to detect emotional cues *Empathetic responses/emotional support absent or forced *Dismissive of pain or anxiety | ( )  Fair | ( )  Adequate: *Responds to emotional cues *Provides empathetic responses/emotional support *Acknowledges pain or anxiety | ( )  Very Good | ( )  Excellent: *Perceptive of emotional cues and encourages emotional expression *Provides empathetic responses/emotional support with genuineness and sincerity *Attentive to pain or anxiety |
| 10.  Respect | ( )  Poor: *Judgmental attitude *Makes you feel inferior | ( )  Fair | ( )  Adequate: *Non-judgmental attitude *Treats you as equal | ( )  Very Good | ( )  Excellent: *Accepting attitude *Establishes partnership |
| 11.  Closure | ( )  Poor: *No explanation of impression or plan *No inquiry into remaining questions *No cordial closing remarks | ( )  Fair | ( )  Adequate: *Explains impression and plan *Inquires about remaining questions *Cordial closing remarks | ( )  Very Good | ( )  Excellent: *Thorough discussion of impression and plan *Seeks unanswered questions, verification of understanding, and comfort level *Warm and grateful closing remarks |

**Comments**

| 12. |
| --- |
