# Supplementary material for: It's Getting Hot in Here: Piloting a Telemedicine OSCE Addressing Menopausal Concerns for Obstetrics and Gynecology Clerkship Students
Source: MedEdPORTAL. 2021 Apr 28;17:11146. doi: 10.15766/mep_2374-8265.11146 (PMC8079425; doi:10.15766/mep_2374-8265.11146)
Supplement: Supplementary file 1 — Preencounter Learner Instructions.docxStandardized Patient Case.docxPreencounter Learner Information (Door Card).docxPostencounter Learner Note Scoring Criteria.docxPostencounter Learner Note (Blank).docxPostencounter Learner Note (Example).docxPostencounter Standardized Patient Checklist.docx [file mep_2374-8265.11146-s001.zip › Educational_Summary_Report_11146.pdf]

MedEdPORTAL® | The AAMC Journal of Teaching and Learning Resources

Original Publication

Open Access

It's Getting Hot in Here: Piloting a Telemedicine OSCE Addressing Menopausal Concerns for Obstetrics and Gynecology Clerkship Students

Hadley W. Reid*, Kelly Branford, Tracey Reynolds, Melody Baldwin, MD, MPH, Sarah Dotters-Katz, MD, MMHPE

*Corresponding author: hwr5@duke.edu

Abstract

Introduction: Although menopause is a common condition, trainees still express high levels of discomfort with managing climacteric symptoms. Trainees also receive little preparation for conducting telemedicine visits, which have become increasingly important in clinical care. We present a formative standardized patient (SP) encounter to introduce medical students to the diagnosis and treatment of menopausal symptoms and the process of conducting a telemedicine visit. **Methods:** We designed a virtual telemedicine encounter with an SP for medical students. Students received feedback via a post-encounter note on history taking, differential diagnosis, and diagnostics/management and an SP debrief. We collected student input on the experience at the midpoint and end of clerkship and analyzed it for recurring themes. We calculated summary statistics from student post-encounter notes. **Results:** Thirty-two OB/GYN students completed the menopause telemedicine SP encounter between April and June 2020. Students scored a median of 20 out of 45 (interquartile range: 18, 22) on the post-encounter note. All students correctly provided a diagnosis of perimenopause/menopause; however, 50% did not offer any strategy for counseling or managing menopausal symptoms. Students expressed discomfort with using a telehealth format (78%) but found it a useful skill to practice (47%). A majority (66%) found the educational encounter to be of excellent or above-average educational value. **Discussion:** While medical students demonstrated discomfort with both managing menopause and utilizing a telemedicine format, this SP case provided an opportunity for them to practice both skills in a safe learning environment. The majority of participants rated the learning experience highly.

Keywords
Standardized Patient, Telemedicine, Telehealth, Objective Structured Clinical Examination, OSCE, Menopause, Women's Health, Case-Based Learning, Online/Distance Learning, Virtual Learning

Educational Objectives

By the end of this activity, learners will be able to:

1. Obtain a complete problem-focused history as it pertains to gynecologic symptoms.
2. Perform an assessment of symptoms associated with hypoestrogenism.
3. Develop a complete differential diagnosis for menopausal symptoms.
4. Document a management plan and counsel a patient regarding management of menopausal symptoms.
5. Demonstrate empathy to the concerns expressed by a patient and increased comfort with conducting a medical interview and establishing therapeutic rapport virtually.

Citation:
Reid HW, Branford K, Reynolds T, Baldwin M, Dotters-Katz S. It's getting hot in here: piloting a telemedicine OSCE addressing menopausal concerns for obstetrics and gynecology clerkship students. *MedEdPORTAL*. 2021;17:11146.
https://doi.org/10.15766/mep_2374-8265.11146

Introduction

As women continue to routinely live into the eighth decade of life, menopause has become a ubiquitous and important issue of middle age for female patients and their providers.[1] However, multiple previous studies have shown that internal medicine, family medicine, and OB/GYN residents are uncomfortable with their level of knowledge on climacteric change and menopause management/treatments. When surveyed, up to 70% of fourth-year OB/GYN residents reported discomfort with topics related to management and sequalae of menopause, and more than 90% of postgraduate trainees in internal medicine, family medicine, and OB/GYN reported feeling unprepared to manage women experiencing menopause.[2,3]

Copyright © 2021 Reid et al. This is an open-access publication distributed under the terms of the Creative Commons Attribution license.

The majority of the existing literature focuses on gaps in knowledge in graduate medical education, but the discomfort with managing and addressing menopause in clinical encounters begins in medical school. The Association of Professors of Gynecology and Obstetrics recommends integration of menopause into OB/GYN clerkship curricula, yet, in practice, medical students may still not gain sufficient experience with the topic.[4] A survey of students at the University of Connecticut School of Medicine found that only 60% reported any exposure to menopause during their OB/GYN clerkship, significantly less than the percentage who reported exposure to relatively less common conditions such as pre-eclampsia.[5] Lack of exposure leads to discomfort in both management and discussion of menopause in clinical encounters.

In addition, the use of telemedicine is becoming increasingly widespread, especially for issues such as menopausal complaints that can be assessed remotely without the need for extensive physical examination. The consulting firm McKinsey & Company estimated that the share of patients in the United States using telehealth increased from 11% in 2019 to 46% in April of 2020.[6] While this change was driven in large part by restrictions on in-person care due to the risks posed by the COVID-19 pandemic, the rapid transition to telemedicine during this time has made clear that it is a viable and important option for care provision going forward. It is therefore vital to increase trainee comfort with virtual health care provision.

Although often lacking in randomized study designs, standardized patient (SP) encounters have been shown to improve learner communication and clinical skills in a variety of clinical fields, including OB/GYN.[7-10] However, the majority of reports on simulation-based learning in OB/GYN have focused on practicing fetal deliveries, acquiring operative skills, or eliciting the sexual history specifically.[9,11-13] To our knowledge, use of SPs has not been reported as a way increase comfort with caring for menopausal women. Furthermore, as health systems continue to explore new methods of care delivery, comfort with using telemedicine for clinical encounters, especially those dealing with personal medical topics such as menopause, is becoming an increasingly important teaching point. Experiences integrating telemedicine into nursing curricula have shown that this improves student comfort using this modality even for complex or sensitive topics such as stroke care.[14,15]

An evidence base exists for the use of simulations and SPs in training students for OB/GYN encounters, which may be especially pertinent when introducing a new interactive skill such as conducting a telehealth encounter. Additionally, given the lack of trainee comfort with discussing and managing menopausal symptoms, an SP encounter, which has been previously used to practice gathering sensitive information such as the sexual history, provides valuable experience.[16] Here, we aim to jointly address this lack of comfort with menopause care and the need for greater telehealth education for medical students by describing the implementation of a virtual telemedicine SP case exploring diagnosis and management of menopause for OB/GYN clerkship students at the Duke University School of Medicine. The exercise was formative, including an evaluation of communication skills in discussing a potentially emotional topic and an assessment of student knowledge of how to evaluate and treat perimenopausal and menopausal patients.

Methods

Target Audience

Medical students at Duke traditionally participate in a 6-week OB/GYN clerkship during their clinical second year. However, given the circumstances related to the COVID-19 pandemic, from April-June 2020 we delivered clerkship didactics, including the National Board of Medical Examiners Shelf Exam, in a condensed virtual manner over 4 weeks before resuming condensed in-person clinical rotations in July 2020. Prior to the clerkship, students were briefly exposed to menopause in the preclinical curriculum through the study of the female hormone axis in their physiology curriculum and as a risk factor for certain conditions in pathology. The OB/GYN clerkship included small-group case-based learning on relevant obstetric or gynecologic issues; however, students had not received instruction on menopause through these didactics prior to the SP encounter.

The encounter occurred at the end of the first week of the 4-week didactic portion of the OB/GYN clerkship. We instructed students that they would perform the telemedicine case with the SP over the course of 30 minutes (Appendix A). The encounter occurred remotely over Zoom. Thirty-two clerkship students participated in the menopausal symptoms telemedicine case.

SP Preparation

An SP educator and an experienced SP initially vetted the case. This process took 2 hours for each individual. The SP educator then trained two SPs for the encounter. The total duration of the training was 3 hours, which comprised 1 hour of home study and 2 hours of online training. The training included discussing details of the script, engaging in extensive role-play, addressing unanswered questions, and practicing verbal feedback. We provided SPs with information about the history they should give in response to student questions and any physical exam

Copyright © 2021 Reid et al. This is an open-access publication distributed under the terms of the Creative Commons Attribution license.

findings they should portray (Appendix B). No additional props or materials were used in this encounter.

Patient Encounter

Groups of 8-10 students rotated through the menopause case over the course of the session; each student had a one-on-one encounter with an SP. We sorted virtual learners with their assigned SP into individual Zoom breakout rooms where they viewed information about the encounter and necessary tasks via screenshare prior to beginning the encounter (Appendix C). Students were informed that their patient, a 51-year-old female named Lynette Springfield, had presented for a telehealth encounter as a new patient with the chief complaint of hot flashes. Students were given 15 minutes for the interview portion of the encounter and any necessary counseling or patient education. In the pilot version of the telehealth encounter, students were provided with vital signs on the virtual door card and were given key physical exam findings (e.g., normal thyroid and nontender, nongravid abdomen). However, due to the telehealth nature of the visit, we subsequently increased the realism of the encounter by not providing vital signs or physical exam findings. The physical exam portion was still worth 4 points in the updated exercise: 2 points for noting that no vital signs were available and 2 points for documenting the patient's general appearance. This change is reflected in the educational materials in Appendix D.

Warnings were given with 5 minutes remaining, as well as when time had elapsed. Sessions were recorded via the Record feature on Zoom. Proctors were available throughout the session, and students were able to send a message to the proctor from their breakout room to indicate that they required assistance.

After the encounter, learners were given 15 minutes to complete a post-encounter note using a provided template (Appendices E and F). After finishing their post-encounter note, students reentered their Zoom breakout room to receive real-time feedback from the SP on their performance. SPs were provided with a checklist (Appendix G) with guidance for feedback on aspects of student interpersonal communication and were instructed to emphasize how the digital environment affected communication.

Students were able to complete the encounter, post-encounter note, and verbal feedback from any location in which they had an internet connection and privacy. Given that this was a formative encounter with a 15-minute time limit to complete the post-encounter note, we did not have significant concerns about students accessing outside resources when completing the note.

Case-Based Learning Module

Following the SP encounter, the topic of menopause was discussed in small-group case-based learning using *Case Files: Obstetrics and Gynecology*[17] as a guide. Clerkship students signed up in advance to lead interactive case presentations on selected cases in *Case Files: Obstetrics and Gynecology,* including "Case 30: Perimenopause." Each student presented the brief clinical case vignette from *Case Files: Obstetrics and Gynecology* and guided their classmates through the differential diagnosis and management of the scenario. A faculty member and student teaching assistant were present at each session to answer questions and clarify concepts related to the course material.

Learner Assessment

Learners participated in the SP case after 1 week of OB/GYN clerkship didactics. Therefore, this exercise was a formative, as opposed to summative, assessment and included verbal feedback from the SP on communication skills and comfort with conducting a telemedicine encounter, as well as an assessment of learners' comfort with diagnosing and managing menopausal symptoms via their written note.

Students received immediate verbal feedback from SPs on their history taking and communication skills, including empathy, rapport building, and perceived comfort in the virtual environment. Additionally, trained OB/GYN teaching assistants reviewed and scored the post-encounter note utilizing a prespecified grading rubric. The teaching assistants were senior medical students. They were oriented to the scoring rubric and the scoring of the example note. Additionally, the OB/GYN clerkship director was available to provide input on any notes in which the scoring was ambiguous. The scoring criteria included the students' differential diagnosis, diagnostic steps, and proposed management. Ultimately these scores were tabulated into a final numeric grade with a maximum score of 45. This score was formative and did not impact the learners' final clerkship grade.

Evaluation

Evaluation of the SP encounter was conducted through analysis of collected student notes. Data from post-encounter learner notes and post-encounter feedback collected from students at mid- and postclerkship reviews were analyzed. Summary statistics of continuous variables and categorical variables were calculated as reported below. Data analysis was performed with R Studio (version 1.2.1335). Midpoint and end-of-clerkship feedback was compiled by the undergraduate medical education team.

Copyright © 2021 Reid et al. This is an open-access publication distributed under the terms of the Creative Commons Attribution license.

Qualitative input on the SP encounter was collected by the clerkship director via student feedback during the midrotation feedback. Specifically, each student was asked, “What did you think of the telemedicine SP encounter?” Quotes from students were recorded in writing and then compiled to determine trends. Additionally, the school of medicine required all students to complete an end-of-rotation survey. Student ratings of how well the SP encounter enhanced their learning and/or identified areas of weakness in their knowledge were collected from the end-of-rotation survey.

Results

Thirty-two clerkship students completed the menopause telehealth encounter between April and June 2020. Eighteen participants (56%) self-identified as male. The median score on the post-encounter note was 20 out of 45 (interquartile range [IQR]: 18, 22). The physical exam median score was 75% (IQR: 50%, 81%), and the differential diagnosis median score was 50% (IQR: 40%, 60%), which were the highest-scoring portions (Table 1). On average, each student correctly identified a median of two leading diagnoses (i.e., those diagnoses considered higher likelihood or “can’t miss”) on their post-encounter note. Students received a median of 6 out of 13 possible points (IQR: 5.75, 7.00) for history taking and 6 out of 18 possible points (IQR: 4.00, 6.25) for diagnostics and management.

All students correctly identified menopause/perimenopause as the most likely diagnosis, and 27 (84%) identified hyperthyroidism as an additional possible diagnosis (Table 2). Students recognized other possible diagnoses at lower rates, including malignancy (28%), anxiety (6%), and pregnancy (34%). Students were largely able to support their proposed diagnoses with appropriate evidence from the clinical history. The diagnosis of hyperthyroidism had the greatest difference between students who correctly placed it on their differential diagnosis (84%) and those who correctly provided supporting evidence (62%).

By far, the weakest section was management and counseling on strategies for menopause management in the assessment and plan. Fifty percent of students did not identify a single appropriate strategy for management of menopausal symptoms. Only seven student notes (22%) listed lifestyle modifications and/or counseled on the use of a selective serotonin reuptake inhibitor or serotonin–norepinephrine reuptake inhibitor. Six learners (19%) suggested use of estrogen vaginal cream. However, only three students (9%) correctly listed use of hormone replacement therapy including progesterone in their plan (Table 3). Overall, only two learners identified three possible management strategies, and no learners identified more than three strategies.

With regard to trends in feedback from students, many acknowledged the difficulty in connecting emotionally with patients over a telemedicine encounter, including difficulty reading/mirroring body language, difficulty expressing empathy, and increased instances of talking over the patient. Of the 32 clerkship students, 25 (78%) mentioned awkwardness with the telemedicine format during their midclerkship feedback, although 15 (47%) appreciated the telemedicine format of the SP encounter as useful practice (Table 4). On the postclerkship survey, a majority of students (66%) described the virtual SP encounter as having excellent or above-average educational value, and 50% felt the encounter enhanced learning extremely well or very well.

Table 2. Differential Diagnosis on Post-encounter Learner Note (*N* = 32)

Differential Diagnosis	No. (%) Correctly Identified	No. (%) Correctly Provided Evidence After Identifying Diagnosis
Menopause/perimenopause	32 (100)	32 (100)
Hyperthyroidism	27 (84)	20 (62)
Pregnancy	11 (34)	9 (28)
Malignancy	9 (28)	9 (28)
Pheochromocytoma	5 (16)	5 (16)
Anxiety	2 (6)	2 (6)
Infection	1 (3)	1 (3)
Alcohol abuse	1 (3)	0 (0)
Dietary side effect	1 (3)	0 (0)

Table 1. Assessment Scores (*N* = 32)

Evaluated Material	Maximum Possible Score	Median Score (Interquartile Range): Points	Median Score (Interquartile Range): Percentage
Overall	45	20 (18.00, 22.00)	44 (40, 49)
History	13	6 (5.75, 7.00)	46 (44, 54)
Physical exam	4	3 (2.00, 3.25)	75 (50, 81)
Differential diagnosis	10	5 (4.00, 6.00)	50 (40, 60)
Diagnostics and management	18	6 (4.00, 6.25)	33 (22, 35)

Table 3. Management of Menopause on Post-encounter Learner Note (*N* = 32)

Management Strategy	Correctly Suggested: No. (%)
Lifestyle modifications	7 (22)
Validation and reassurance	3 (9)
Selective serotonin reuptake inhibitor/serotonin–norepinephrine reuptake inhibitor	7 (22)
Hormone replacement therapy with progesterone	3 (9)
Estrogen cream	6 (19)
Did not correctly suggest any management strategies	16 (50)

Copyright © 2021 Reid et al. This is an open-access publication distributed under the terms of the Creative Commons Attribution license.

Table 4. Descriptive Feedback on Telemedicine Encounter ($N = 32$)

Theme	No. (%) Responding	Example Comments
Awkwardness with telemedicine format	25 (78)	"Awkward, feel the distance, harder to show and relay empathy—figuring out the camera and looking angle, how to make eye contact."<br>"Body language is challenging online."
Helpful to practice telemedicine	15 (47)	"Important skill to develop that we had not experienced yet, good learning exercise."<br>"New form of communication, new tool—appreciate exposure now—tips are helpful, great to practice this."
Technical difficulties	3 (9)	"Internet lag."
Not too different	6 (19)	"Format close to typical."
Menopausal symptom content useful/challenging	4 (13)	"Reasonable case, common and important."

Discussion

To address the gap in medical education for training in both telemedicine and diagnosis and management of menopause, we designed and executed a telemedicine encounter with an SP focused on climacteric symptoms. The case included a virtual telehealth encounter with 15 minutes for history taking with the SP and 15 minutes to complete a post-encounter note. Students received informal verbal feedback on communication, empathy, rapport building, and ease with the virtual environment from the SP at the conclusion of the exercise.

To our knowledge, this is the first SP exam or OSCE to address menopause through telemedicine. Previous published attempts to increase trainee comfort with delivery of care through telemedicine have focused on residents.[18,19] However, the feedback we gathered from students indicated that there is an appreciation for introducing this topic earlier in training. It is also striking to note that 78% of our student sample experienced awkwardness with the online format. This is a significantly higher percentage than in the one other instance we could find of a telemedicine OSCE delivered to medical students. Cantone and colleagues reported that only 23 out of 231 comments were coded as finding the experience "important and/or difficult, challenging but educational."[20] Our results may in part be due to the sensitive nature of the discussion of menopause and students' minimal prior didactic experience on the topic, although this experience did occur in the latter part of the students' clinical year. Because of the condensed nature of the curriculum being delivered after the virtualization of all clerkships at our institution due to COVID-19, as well as our desire to offer students practice with and formative feedback from an SP early in their clerkship, we were unable to give didactics on menopause prior to the SP encounter. In replicating this experience, educators could consider integrating it later in the clerkship, after students have received didactic content on menopause care, especially if it is to be used as a summative evaluation. Regardless, the discomfort with telehealth reported by our sample should be an impetus to exposing students to telemedicine early in their clinical careers due to the steep learning curve for using it effectively.

The evaluation of students' notes revealed patterns similar to previous reports in the literature. All students in this group were able to correctly diagnose menopause and provide supporting evidence. However, they had much greater difficulty validating patient concerns and suggesting appropriate management. Our medical students were not alone in this; postgraduate trainees in internal medicine, family medicine, and OB/GYN have also reported discomfort with management of menopause.[3] This virtual SP encounter provides a venue for students to practice and identify gaps in menopause-related knowledge and to increase student comfort with these aspects of menopause care. This SP encounter could also be used with graduate-level trainees to increase comfort in this area. We opted for immediate formative SP verbal feedback on learner communication, empathy, and rapport building, which has previously been highly rated by students for its intimate and personalized nature.[21] However, this exercise could also be performed with a written evaluation of learner performance if a more-formal evaluation is desired, for instance, by distributing a completed SP checklist to students after the encounter.

The virtual nature of the telemedicine case has several other advantages. It can be performed from any location and decreases the need for expensive in-house simulation centers and personnel. Similarly, the need to be in person is also mitigated by this format; thus, for medical school clerkships that have students distributed across multiple clinical sites, it can be used without requiring students to return to a central campus while still providing equitable experiences across sites. Additionally, many virtual technologies such as Zoom, which we used in this case, have a built-in feature to allow the patient encounter to be recorded for student viewing and self-evaluation. Finally, although we used trained SPs, the case could also be adapted to paired student or resident role-play if the involvement of trained SPs is not feasible.

Copyright © 2021 Reid et al. This is an open-access publication distributed under the terms of the Creative Commons Attribution license.

Limitations

This study had several limitations. First, we had a relatively small sample size of 32 students going through the pilot iteration of this telemedicine encounter on menopausal symptoms. However, we believe their experience with the encounter should still be representative. Additionally, due to limitations in the timing of our curriculum, students participated in the encounter prior to receiving didactics on the diagnosis and management of menopause, which limited our ability to use this exercise to measure the effectiveness of didactics on menopause on student knowledge. As the telemedicine SP encounter was formative in nature, this sequence was still appropriate for our students to highlight gaps in knowledge and opportunities to improve communication and rapport building in a virtual format, but this could be altered in future iterations of the exercise. Finally, we did not document the verbal student feedback from SPs at the end of the encounter as it was meant as an informal opportunity to discuss student strengths and weaknesses in communication. In the future, this feedback could be documented and analyzed to gain another data point to monitor student progress and more formally assess student comfort with telehealth.

Conclusions

Overall, we found the use of telemedicine for an SP case with an ultimate diagnosis of menopause to be successful in both introducing students to the process of conducting a virtual patient encounter and familiarizing them with the principles of diagnosing and treating climacteric change in their OB/GYN clerkship. We explored menopause in the context of an OB/GYN curriculum, but given the common nature of related climacteric complaints, the case is relevant to a variety of specialties, including internal medicine and family medicine. As we continue to move toward a more virtual world, the ability to address common complaints such as menopause through telemedicine should be integrated into the clinical curriculum early on to prepare the next generation of trainees.

Appendices

A. Preencounter Learner Instructions.docx

B. Standardized Patient Case.docx

C. Preencounter Learner Information (Door Card).docx

D. Postencounter Learner Note Scoring Criteria.docx

E. Postencounter Learner Note (Blank).docx

F. Postencounter Learner Note (Example).docx

G. Postencounter Standardized Patient Checklist.docx

All appendices are peer reviewed as integral parts of the Original Publication.

Hadley W. Reid: Third-Year Medical Student, Duke University School of Medicine; ORCID: https://orcid.org/0000-0002-4329-7831

Kelly Branford: Director of Clinical Skills Program, Office of Curricular Affairs, Duke University School of Medicine

Tracey Reynolds: Standardized Patient Coordinator, Office of Curricular Affairs, Duke University School of Medicine

Melody Baldwin, MD, MPH: Assistant Professor, Department of Obstetrics and Gynecology, Duke University Medical Center

Sarah Dotters-Katz, MD, MMHPE: Assistant Professor, Department of Obstetrics and Gynecology, Duke University Medical Center

Disclosures

None to report.

Funding/Support

Research reported in this publication was supported by the National Center for Advancing Translational Sciences of the National Institutes of Health under award number TL1 TR002555.

Ethical Approval

Reported as not applicable.

Disclaimer

The content is solely the responsibility of the authors and does not necessarily represent the official views of the National Institutes of Health.

References

1. Xu J, Murphy SL, Kochanek KD, Arias E. *Mortality in the United States, 2018*. National Center for Health Statistics; 2020. NCHS Data Brief no. 355.

2. Christianson MS, Ducie JA, Altman K, Khafagy AM, Shen W. Menopause education: needs assessment of American obstetrics and gynecology residents. *Menopause*. 2013;20(11):1120-1125. https://doi.org/10.1097/GME.0b013e31828ced7f

3. Kling JM, MacLaughlin KL, Schnatz PF, et al. Menopause management knowledge in postgraduate family medicine, internal medicine, and obstetrics and gynecology residents: a cross-sectional survey. *Mayo Clin Proc*. 2019;94(2):242-253. https://doi.org/10.1016/j.mayocp.2018.08.033

Copyright © 2021 Reid et al. This is an open-access publication distributed under the terms of the Creative Commons Attribution license.

4. APGO Medical Student Educational Objectives for students. Association of Professors of Gynecology and Obstetrics. 2019. Accessed April 28, 2020. https://apgo.org/page/msostudent

5. Schnatz PF, Marakovits K. The next generation of menopause providers: are medical students being properly prepared? *Menopause*. 2008;15(5):871-874. https://doi.org/10.1097/gme.0b013e31816429f8

6. Bestsennyy O, Gilbert G, Harris A, Rost J. Telehealth: a quarter-trillion-dollar post-COVID-19 reality? McKinsey & Company. May 29, 2020. Accessed August 26, 2020. https://www.mckinsey.com/industries/healthcare-systems-and-services/our-insights/telehealth-a-quarter-trillion-dollar-post-covid-19-reality#

7. May W, Park JH, Lee JP. A ten-year review of the literature on the use of standardized patients in teaching and learning: 1996–2005. *Med Teach*. 2009;31(6):487-492. https://doi.org/10.1080/01421590802530898

8. Lane C, Rollnick S. The use of simulated patients and role-play in communication skills training: a review of the literature to August 2005. *Patient Educ Couns*. 2007;67(1-2):13-20. https://doi.org/10.1016/j.pec.2007.02.011

9. Le Lous M, Simon O, Lassel L, Lavoue V, Jannin P. Hybrid simulation for obstetrics training: a systematic review. *Eur J Obstet Gynecol Reprod Biol*. 2020;246:23-28. https://doi.org/10.1016/j.ejogrb.2019.12.024

10. Rodriguez AN, Fisher J, Broadfoot K, Hurt KJ. A challenging obstetric communication experience for undergraduate medical education using standardized patients and student self-reflection. *MedEdPORTAL*. 2015;11:10121. https://doi.org/10.15766/mep_2374-8265.10121

11. Coverdale JH, Balon R, Roberts LW. Teaching sexual history-taking: a systematic review of educational programs. *Acad Med*. 2011;86(12):1590-1595. https://doi.org/10.1097/ACM.0b013e318234ea41

12. Alphonso A, Pathy S, Bruno C, et al. Shoulder dystocia and neonatal resuscitation: an integrated obstetrics and neonatology simulation case for medical students. *MedEdPORTAL*. 2017;13:10594. https://doi.org/10.15766/mep_2374-8265.10594

13. Appleton S, Huguelet P. Laparoscopy skills simulation for the obstetrics and gynecology resident. *MedEdPORTAL*. 2016;12:10460. https://doi.org/10.15766/mep_2374-8265.10460

14. Rutledge CM, Haney T, Bordelon M, Renaud M, Fowler C. Telehealth: preparing advanced practice nurses to address healthcare needs in rural and underserved populations. *Int J Nurs Educ Scholarsh*. 2014;11(1):1-9. https://doi.org/10.1515/ijnes-2013-0061

15. Erickson CE, Fauchald S, Ideker M. Integrating telehealth into the graduate nursing curriculum. *J Nurse Pract*. 2015;11(1):e1-e5. https://doi.org/10.1016/j.nurpra.2014.06.019

16. Hagey JM, Toole J, Branford K, Reynolds T, Livingston E, Dotters-Katz SK. Understanding sexual complaints and history taking: a standardized patient case on dyspareunia for obstetrics and gynecology clerkship students. *MedEdPORTAL*. 2020;16:11001. https://doi.org/10.15766/mep_2374-8265.11001

17. Toy EC, Baker B III, Ross PJ, Jennings JC. *Case Files: Obstetrics and Gynecology*. 5th ed. McGraw Hill Education; 2016:298-305.

18. Sartori DJ, Olsen S, Weinshel E, Zabar SR. Preparing trainees for telemedicine: a virtual OSCE pilot. *Med Educ*. 2019;53(5):517-518. https://doi.org/10.1111/medu.13851

19. Lawrence K, Hanley K, Adams J, Sartori DJ, Greene R, Zabar S. Building telemedicine capacity for trainees during the novel coronavirus outbreak: a case study and lessons learned. *J Gen Intern Med*. 2020:35(9):2675-2679. https://doi.org/10.1007/s11606-020-05979-9

20. Cantone RE, Palmer R, Dodson LG, Biagioli FE. Insomnia telemedicine OSCE (TeleOSCE): a simulated standardized patient video-visit case for clerkship students. *MedEdPORTAL*. 2019;15:10867. https://doi.org/10.15766/mep_2374-8265.10867

21. Newcomb AB, Duval M, Bachman SL, Mohess D, Dort J, Kapadia MR. Building rapport and earning the surgical patient's trust in the era of social distancing: teaching patient-centered communication during video conference encounters to medical students. *J Surg Educ*. 2021;78(1):336-341. https://doi.org/10.1016/j.jsurg.2020.06.018

Received: October 9, 2020
Accepted: February 18, 2021
Published: April 28, 2021

Copyright © 2021 Reid et al. This is an open-access publication distributed under the terms of the Creative Commons Attribution license.
